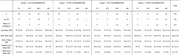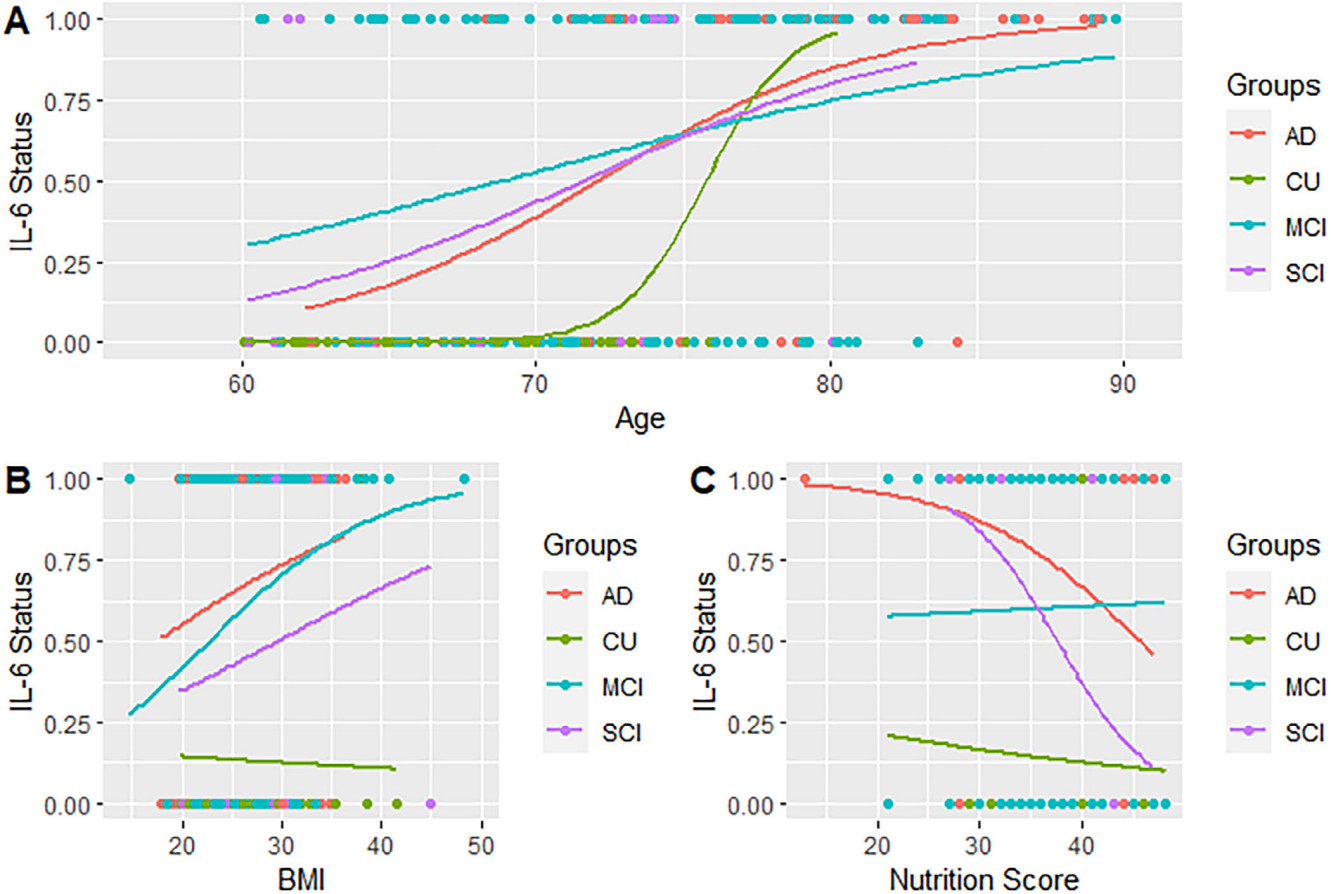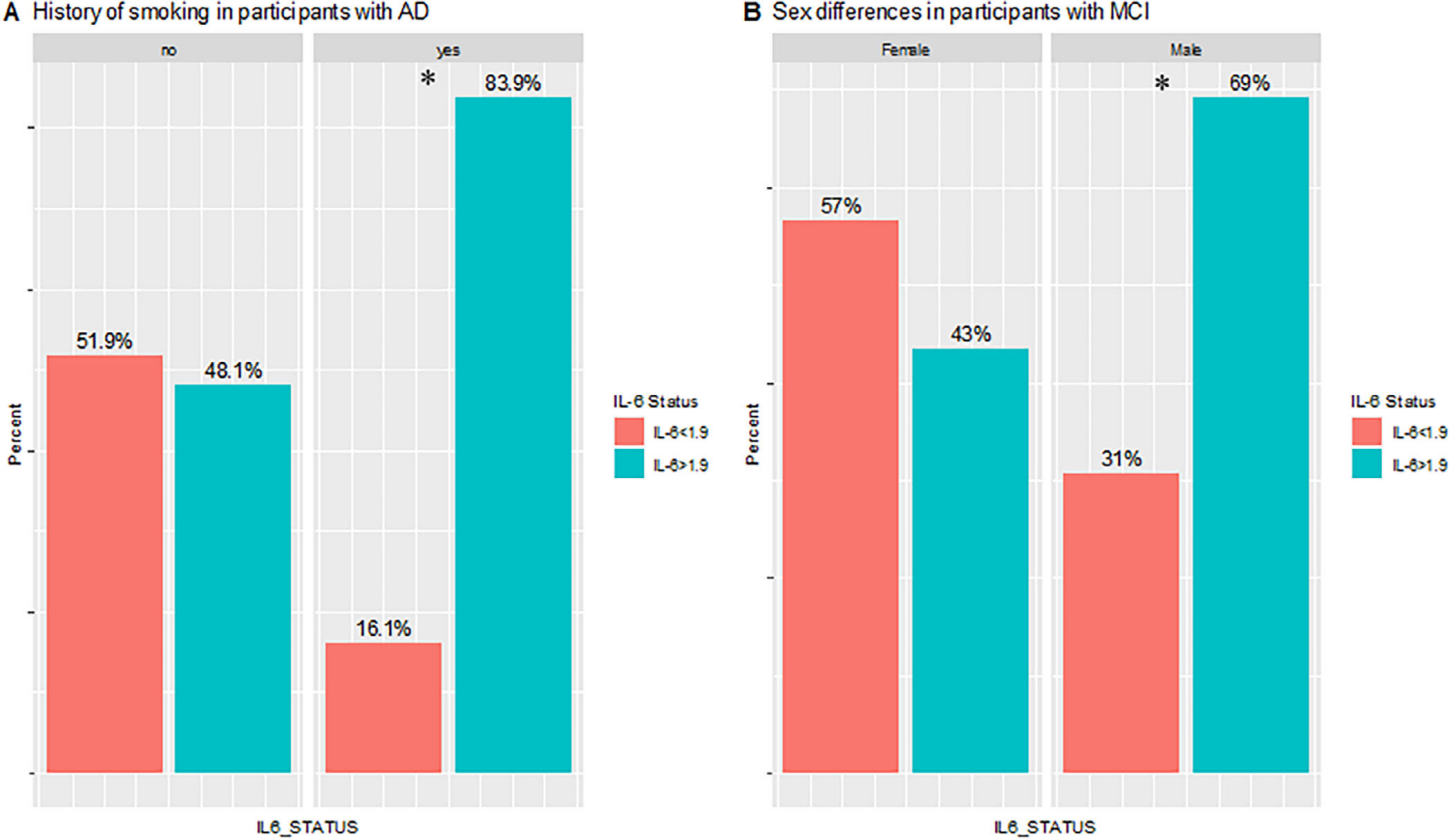# Peripheral Inflammation subgroups in Alzheimer’s disease and related dementias

**DOI:** 10.1002/alz.090393

**Published:** 2025-01-03

**Authors:** Bruna Seixas Lima, Pedro Rosa‐Neto, Natalie Phillips, Michael Borrie, Carlos Tyler Roncero, Durjoy Lahiri, Dvir Dori, Howard Chertkow

**Affiliations:** ^1^ Rotman Research Institute, Toronto, ON Canada; ^2^ Translational Neuroimaging Laboratory, The McGill University Research Centre for Studies in Aging, Montréal, QC Canada; ^3^ Concordia University, Montreal, QC Canada; ^4^ Division of Geriatric Medicine, Western University, London, ON Canada; ^5^ Baycrest/Rotman Research Institute, Toronto, ON Canada; ^6^ Rotman Research Institute‐Baycrest, TORONTO, ON Canada; ^7^ Canadian Consortium on Neurodegeneration in Aging (CCNA), Montreal, QC Canada

## Abstract

**Background:**

A growing body of research has focused on inflammation as both a potential biomarker and a risk factor for Alzheimer’s disease (AD). The cytokine Interleukin‐6 (IL‐6) is involved in the pathogenesis of inflammatory disorders and in the physiological homeostasis of neural tissue. AD has been associated with increased IL‐6 expression in brain, however, increased levels of IL‐6 have also been linked to conditions such as diabetes and hypertension. We examined groups of individuals with AD and related disorders (ADRD) as well as healthy controls to check if elevated levels of IL‐6 (above 1.9 ng/L) were related to the presence of comorbidities.

**Methods:**

We investigated individuals with AD (n = 58); mild cognitive impairment (MCI; n = 139); subjective cognitive impairment (SCI; n = 28); and cognitively unimpaired age matched controls (CU; n = 53). We considered four possible subgroups: Group 0 (defined as individuals with normal IL‐6 levels and without comorbidities that could be linked to elevated IL‐6); Group 1 (individuals with normal levels of IL‐6, despite having comorbidities); Group 2 (individuals with elevated IL‐6 without comorbidities), and Group 3 (individuals with elevated IL‐6 and comorbidities). The comorbidities investigated were hypertension, diabetes, peripheral vascular disease, history of cerebrovascular accident, and rheumatoid arthritis. Logistic regression was used to investigate the relationship between the occurrence of elevated IL‐6, age, sex, history of smoking, body mass index (BMI), Montreal Cognitive Assessment scores (MoCA), nutrition and sleep.

**Results:**

We found individuals with and without elevated IL‐6, and with and without comorbidities in all cohorts (Table 1). We found a significant relationship between elevated IL‐6 and aging in all cohorts (Figure 1a). A significant relationship was also found between elevated IL‐6 and greater BMI (MCI group only; Figure 1b), lower nutrition scores (SCI group only, Figure 1c), history of smoking (AD group only; Figure 2a) and sex, whereby males had more elevated IL‐6 (MCI group; Figure 2b).

**Conclusions:**

We found variation in patterns of peripheral inflammation and other common conditions in aging and ADRD. These variations may reflect different mechanistic groups which might help the understanding of variation in clinical features of ADRD, disease progression and response to different therapeutic interventions.